# 786. Effect of Nirmatrelvir/Ritonavir versus Placebo on COVID-19─Related Hospitalizations and Other Medical Visits

**DOI:** 10.1093/ofid/ofac492.047

**Published:** 2022-12-15

**Authors:** Jennifer Hammond, Heidi Leister-Tebbe, Annie Gardner, Paula Abreu, Weihang Bao, Wayne Wisemandle, Wajeeha Ansari, Jesus Abraham Simón Campos, Rienk Pypstra, James M Rusnak

**Affiliations:** Pfizer Inc, Collegeville, PA; Pfizer Inc, Collegeville, PA; Pfizer Inc, Collegeville, PA; Pfizer Inc, Collegeville, PA; Pfizer Inc, Collegeville, PA; Pfizer Inc, Collegeville, PA; Pfizer Inc., Wyckoff, New Jersey; Köhler & Milstein Research/Hospital Agustín O’Horán, Mérida, Yucatan, Mexico; Pfizer Inc, Collegeville, PA; Pfizer Inc, Collegeville, PA

## Abstract

**Background:**

Nirmatrelvir coadministered with ritonavir (nirmatrelvir/r) is a COVID-19 treatment. This study evaluated nirmatrelvir/r in nonhospitalized, symptomatic adults with COVID-19 at high risk of progressing to severe disease. We report secondary efficacy endpoints associated with COVID-19─related medical visits, including hospitalization details and oxygen support, as of the primary completion data cutoff (Dec 11, 2021).

**Methods:**

In this phase 2/3 double-blind, interventional study, adults with confirmed SARS-CoV-2 and symptom onset ≤ 5 days (d) were randomized 1:1 to receive nirmatrelvir/r 300 mg/100 mg or placebo (PBO) orally every 12 hours for 5 d. COVID-19─related medical visits were collected through Day 28. Oxygen support for COVID-19 and details of COVID-19─related hospitalization, including duration, intensive care unit (ICU) status, and mechanical ventilation, were assessed.

**Results:**

Of the 2246 patients (pts) enrolled globally from Jul to Dec2021, 2085 (nirmatrelvir/r, n=1039; PBO, n=1046) started treatment and met criteria for the modified intent-to-treat population (mITT1; ≤ 5 d of symptom onset, did not/not expected to receive a mAb). Fewer overall COVID-19-related medical visits were reported with nirmatrelvir/r vs PBO (**Table 1**). In addition to fewer hospitalizations being reported with nirmatrelvir/r (n=8 [0.8%]) vs PBO (n=65 [6.2%]), pts receiving nirmatrelvir/r had fewer hospitalized d (Table 2), with mean durations of 9.6 (range, 5.0, 16.0) d with nirmatrelvir/r and 11.2 (range, 2.0, 57.0) d with PBO in hospitalized pts. No pts in the nirmatrelvir/r group and 9 pts (0.9%) in PBO group were admitted to the ICU. No pts in the nirmatrelvir/r group received mechanical ventilation vs 3 pts in the PBO group. Fewer other COVID-19─related nonhospital medical visits were reported with nirmatrelvir/r vs PBO (**Table 3**). In the full analysis set, fewer pts required oxygen therapy for COVID-19 with nirmatrelvir/r (n=9/1120 [0.8%]) vs PBO (n=54/1126 [4.8%]).

**Conclusion:**

High-risk adults with symptomatic COVID-19 treated with nirmatrelvir/r within 5 d of symptom onset had fewer COVID-19─related medical visits and reduced healthcare utilization (no ICU visits, no mechanical ventilation, fewer days in hospital) vs pts receiving PBO. Clinical Trial: NCT04960202.

**Disclosures:**

**Jennifer Hammond, PhD**, Pfizer Inc: Employee|Pfizer Inc: Stocks/Bonds **Heidi Leister-Tebbe, BSN**, Pfizer Inc: Employee|Pfizer Inc: Stocks/Bonds **Annie Gardner, MPH, MSPT**, Pfizer Inc: Employee|Pfizer Inc: Stocks/Bonds **Paula Abreu, PhD**, Pfizer Inc: Employee|Pfizer Inc: Stocks/Bonds **Weihang Bao, PhD**, Pfizer Inc: Employee|Pfizer Inc: Stocks/Bonds **Wayne Wisemandle, MA**, Pfizer Inc: Employee|Pfizer Inc: Stocks/Bonds **Wajeeha Ansari, MPH**, Pfizer Inc.: Stocks/Bonds **Rienk Pypstra, MD, MBA**, Pfizer Inc: Employee|Pfizer Inc: Stocks/Bonds **James M Rusnak, MD, PhD**, Pfizer Inc: Employee|Pfizer Inc: Stocks/Bonds.

